# Mechanistic Insights on the Inhibition of C5 DNA Methyltransferases by Zebularine

**DOI:** 10.1371/journal.pone.0012388

**Published:** 2010-08-24

**Authors:** Christine Champion, Dominique Guianvarc'h, Catherine Sénamaud-Beaufort, Renata Z. Jurkowska, Albert Jeltsch, Loïc Ponger, Paola B. Arimondo, Anne-Laure Guieysse-Peugeot

**Affiliations:** 1 MNHN CNRS UMR7196, Paris, France; 2 INSERM U565, Paris, France; 3 Laboratoire des Biomolécules, UPMC Université Paris 06, CNRS, ENS, FR, Paris, France; 4 Jacobs University Bremen, School of Engineering and Science, Bremen, Germany; University Paris 7, France

## Abstract

In mammals DNA methylation occurs at position 5 of cytosine in a CpG context and regulates gene expression. It plays an important role in diseases and inhibitors of DNA methyltransferases (DNMTs)—the enzymes responsible for DNA methylation—are used in clinics for cancer therapy. The most potent inhibitors are 5-azacytidine and 5-azadeoxycytidine. Zebularine (1-(β-D-ribofuranosyl)-2(1H)- pyrimidinone) is another cytidine analog described as a potent inhibitor that acts by forming a covalent complex with DNMT when incorporated into DNA. Here we bring additional experiments to explain its mechanism of action. First, we observe an increase in the DNA binding when zebularine is incorporated into the DNA, compared to deoxycytidine and 5-fluorodeoxycytidine, together with a strong decrease in the dissociation rate. Second, we show by denaturing gel analysis that the intermediate covalent complex between the enzyme and the DNA is reversible, differing thus from 5-fluorodeoxycytidine. Third, no methylation reaction occurs when zebularine is present in the DNA. We confirm that zebularine exerts its demethylation activity by stabilizing the binding of DNMTs to DNA, hindering the methylation and decreasing the dissociation, thereby trapping the enzyme and preventing turnover even at other sites.

## Introduction

Cancer cells show a highly disturbed epigenetic landscape, which often features a global hypomethylation of the genome that induces abnormal expression of genes and a local hypermethylation of promotors that silences tumor suppressor genes (TSG) [1], [2]. DNA methylation is catalyzed by a family of enzymes called DNA methyltransferases (DNMTs) and occurs in mammals only at position 5 of cytosines in CpG dinucleotides [3]. All DNMTs use S-adenosyl-L-methionine (AdoMet) as methyl group donor. A key feature of the catalytic mechanism of DNMTs is a nucleophilic attack of the enzyme on the carbon-6 of the target cytosine. This attack is performed by the thiol group of the cysteine residue of a conserved Proline-Cysteine-Glutamine (PCQ) motif in the active site of DNMTs and is coupled with protonation of N3 to yield an activated enamine intermediate [4], [5]. This electron flow back into the pyrimidine ring leads to activation of the C5 atom towards electrophilic attack and thus to the addition of the methyl group from the cofactor AdoMet to the cytosine. This step is followed by elimination of the C5 proton and resolution of the covalent intermediate. DNMTs are responsible for *de novo* DNA methylation as well as maintenance of methylation. In eukaryotes different DNMT families are described; DNMT1 is known as the maintenance methyltransferase, since it preferentially binds and methylates hemimethylated DNA; whereas DNMT3a and 3b act as *de novo* methyltransferases. DNMT3L, required for the establishment of maternal genomic imprints, lacks the catalytic activity and participates in *de novo* methylation through stimulation of DNMT3a [6].

**Figure 1:**
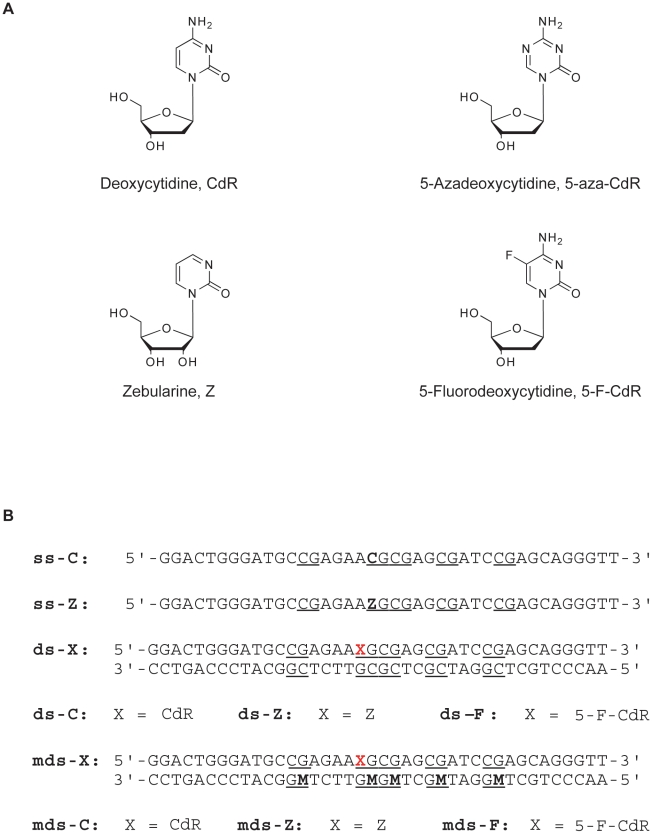
Structure of the inhibitors and sequence of the duplexes used in this study. (A) Nucleosides used to trap cytosine-5 methyltransferases (DNMTs) in comparison to deoxycytidine. (B) Sequences of the oligonucleotides: CdR indicates deoxycytidine, Z indicates zebularine, 5-F-CdR indicates 5-fluorodeoxycytidine, M indicates 5-methyldeoxycytidine, ss single-stranded DNA, ds double-stranded DNA, mds hemimethylated DNA duplex. The CpG site are underlined.

Inactivation of DNMTs is the most effective way of inhibiting DNA methylation and, thereby, removing the hypermethylation of TSG promoters in cancer cells [7]. Therefore, many DNA methylation inhibitors have been developed. Among them, 5-azacytidine (5-aza-CR, Vidaza®) and 5-azadeoxycytidine (5-aza-CdR, Dacogen®) (Fig. 1) have gained FDA approval for the treatment of myelodysplastic syndrome, a preleukemic bone marrow disorder [8], [9]. These compounds are cytidine analogues that once incorporated into the DNA covalently trap the DNMTs on the DNA by forming a suicide complex. After DNMT binding to the C6 of the 5-aza-CdR incorporated into the DNA, methyl group transfer will occur, but no H is present on the N5, which precludes the resolution of the complex [10], [11]. As a result of the irreversibility of the covalent complex, further methylation of cytosine residues is inhibited [12], [13], leading to a massive loss of DNA methylation [14]. The resulting hypomethylation of the genome has been associated with the activation of certain genes previously silenced [15], [16] and among them TSG [17].

One disadvantage of the azanucleosides is their instability in aqueous solutions [18], [19], but this can be overcomed by the use of other more stable analogues, such as zebularine or 5 fluoro-deoxycytidine (5-F-CdR) (Fig. 1A), which also inhibit DNA methylation after incorporation into DNA [17]. 5-F-CdR has antitumor and demethylating properties [20] and, as 5-aza-CdR, once incorporated in DNA traps covalently the DNMT [21]. After C6 attack and transfer of the methyl group, no β-elimination can occur with release of the enzyme, because of the presence of the fluoro on C5 [22].

Zebularine [1-(β-D-ribofuranosyl)-2(1H)-pyrimidinone], a cytidine lacking the 4-amino group, is the most recent addition to the list of demethylating agents in the family of nucleoside analogues (Fig. 1). It was first synthesized in 1961 and characterized as a potent inhibitor of cytidine deaminase with antitumor properties [23]–[25]. It was then described as a DNMT inhibitor [26]–[29], inhibiting DNA methylation [30] and reactivating silenced genes [31]–[33] similarly to 5-aza-CdR. Moreover, zebularine enhances tumor cell chemo- and radiosensitivity [34] and has antimitogenic and angiostatic activities [35], [36]. Additionally, zebularine is very stable and has a half-life of ca. 44 h at 37°C at pH 1.0 and ca. 508 h at pH 7.0, making oral administration of the drug possible. Zebularine is also minimally cytotoxic *in vitro* and *in vivo* [31]. Its low toxicity allowed it to be given continuously at low dose to maintain demethylation for a prolonged period [37]. When 5-aza-CR and 5-aza-CdR treatments are arrested, DNA is remethylated. However, when cancer cells are transiently treated with 5-aza-CdR and then subjected to a continuous treatment with zebularine, remethylation is hindered and gene expression is maintained, suggesting that a combinatorial treatment method gives a better response in the inhibition of DNA methylation [37]–[39]. However, zebularine is not without drawbacks and further study is necessary to fully understand the clinical effects it will have on cancer patients. For example, higher concentrations of zebularine are needed to obtain similar levels of demethylation in cells in comparison with 5-aza-CdR [40], [41]. Moreover zebularine suppresses the apoptotic potential of 5-fluorouracil against human oral squamous cell carcinoma cells, indicating that combination therapies have to be carefully investigated [42]. It has also been shown that zebularine is a potent mutagen in *Escherichia coli* [43].

Because of its potential, we were interested in elucidating the mechanism of action of the drug. Recently, van Bemmel et al. [44] have compared the interaction of zebularine-containing DNA with bacterial M.*Hha I* and mammalian DNMT1 to 5-aza-CR-containing DNA. They observe similar inhibition rates while the ternary complex differs: 5-aza-CR forms an irreversible complex, zebularine instead forms a reversible covalent complex. Accordingly, this difference may account for the differing cytotoxicity observed between the two inhibitors. Here, we further characterize the complex formed by zebularine with bacterial and mammalian DNMTs and propose a hypothesis for the increased stability of the reversible covalent complex. In this study, we have chosen to use 5-F-CdR as comparison instead of the 5-aza derivatives for two reasons. First, 5-F-CdR was shown, as 5-aza-CdR or 5-aza-CR, to trap covalently the DNMTs once incorporated in the DNA [21]. Second, this nucleoside is stable and commercially available, whereas 5-aza-CdR is instable in aqueous solution and cannot be obtained commercially incorporated into DNA. First, the DNA binding affinity of the tested DNMTs is increased when zebularine is incorporated in the DNA duplex compared to cytidine and 5-fluorodeoxycytidine. Second we detect no methylation on zebularine. The absence of the 4-amino group seems to prevent the activation of the cytosine C5 position and thus the methyl group transfer. Third we fail to observe an irreversible covalent complex by SDS-PAGE gel, in agreement with the observation of van Bemmel *et al.* [44]. Fourth we show that the ternary complex with AdoMet, DNMT and DNA containing zebularine is much more stable than the one formed with unmodified DNA. The lack of zebularine methylation can explain this stabilization as it has been described that the steric clash due to the methylation of position 5 of the base induces the dissociation of the DNA/enzyme complex [45].

## Results

### Zebularine increases the DNA binding of DNMTs

We first analyze the capacity of zebularine-containing DNA (ds-Z, Fig. 1B) to bind to the bacterial DNA methylase M.SssI. M.SssI is used as a model system since it is the only known prokaryotic DNMTs, which recognizes the short sequence CG, and thus has the same specificity as mammalian DNMTs. In addition, it contains all ten conserved motifs of C5-DNMTs [46]. Duplexes ds-Z (containing zebularine) is compared to duplexes ds-C (containing cytidine) and ds-F (containing 5-F-CdR).

**Figure 2:**
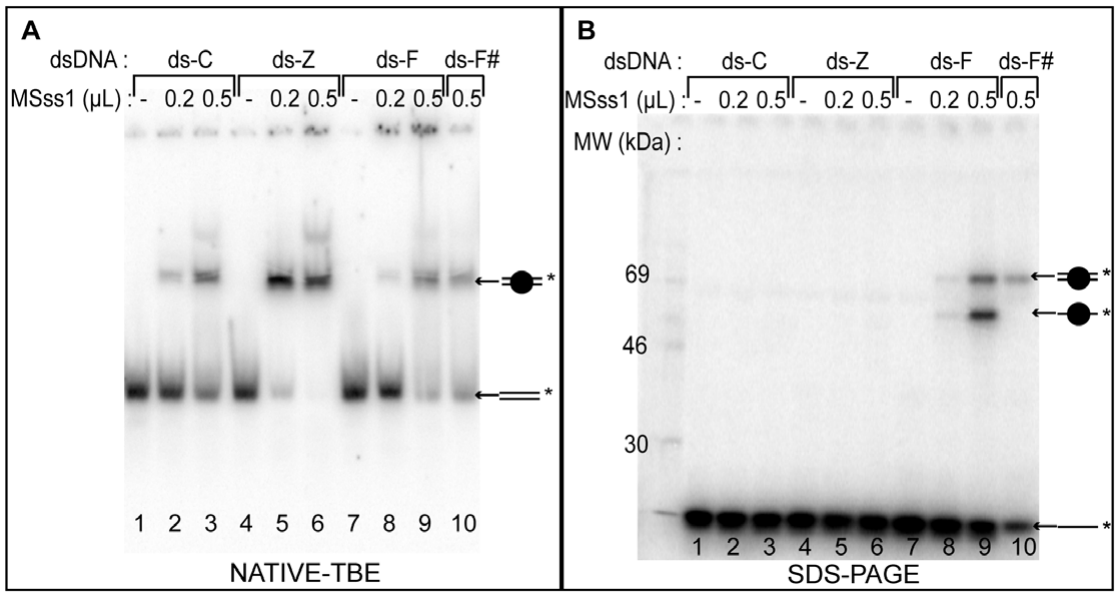
DNA binding and cross-linking of *M.SssI* by gel shift and SDS-PAGE analysis. 5′ [γ^32^P]-labeled duplexes on the stranded containing the modified base (ds-Z, ds-F and the control ds-C) or on the complementary strand (ds-F^#^) were incubated with 0.2 µL (46 nM), 0.5 µL (115 nM) or without M.SssI as mentioned on the top of the gel for 16 h at 16°C and loaded onto a 4% non-denaturing TBE 1X gel (A) or onto a 10% SDS PAGE (B). Schematic representation of the labeled single-stranded DNA (––*) or ds ( = *) are indicated on the right of the gel and the circle represents the enzyme bound to labeled single-stranded or double-stranded DNA.

The duplexes are incubated with M.SssI (10 and 25 fold excess) in the buffer supplied by NEB, supplemented with 300 µM of AdoMet and 100 µg/mL of BSA. Incubations are carried out for 16 h at 16°C as Shigdel and He demonstrated by a time-course experiment that it takes 16 h at 16°C to obtain efficient irreversible cross-linking between a probe containing 5-F-CdR and the bacterial methyltransferase M.HhaI [47]. We confirm that the cross-linking efficacy between M.SssI and duplexe ds-F is higher at 16°C than at 37°C. Half of the reaction mixtures was loaded onto a 4% native TBE polyacrylamide gel (Fig. 2A), and the other half on a 10% denaturing SDS-PAGE after boiling in Laemmli buffer (Fig. 2B). In the native TBE gel (Fig. 2A), we can see that M.SssI efficiently binds modified (ds-Z and ds-F) and non modified (ds-C) duplexes as shown by the retarded band. To determine the C_50_, concentration at which 50% of complex is formed, we measured M.SssI binding on the three duplexes using 7 dose levels of M.SssI from 11 nM to 230 nM (Fig. S1). We find a C_50_ of 60±4 nM for ds-C (mean of two experiments), 46±7 nM for ds-Z (mean of four experiments) and 63±1 nM for ds-F (mean of two experiments). This result clearly shows that zebularine increases the affinity of the enzyme for the duplex and it is more efficient than 5-fluorodeoxycytidine.

### Zebularine failed to form an irreversible covalent complex with DNMT

The loading of the samples on a denaturing 10% SDS gel after boiling allows to determine the nature of the covalent complex formed between zebularine-containing DNA and M.SssI. As expected, the negative control ds-C did not show any cross-linking (Fig. 2B, lanes 2 and 3). Interestingly, we fail to observe the formation of a covalent complex between M.SssI and ds-Z (Fig. 2B, lanes 5 and 6). This result is in agreement with the observations reported by van Bemmel *et al.* [44]. They observed by SDS-PAGE gel analysis that the ternary complex between a ds-DNA containing zebularine and M.HhaI is reversible and heat labile.

As a positive control, we use ds-F that cross-links efficiently to M.SssI, as shown by the appearance of two bands with retarded mobility on the SDS-PAGE gel (Fig. 2B, lanes 8 and 9). The upper band has a mobility of approximately 68 kDa corresponding to M.SssI (42 kDa) bound to the duplex (26 kDa). The lower band has a mobility of approximately 55 kDa, matching the molecular weight of the single-stranded oligonucleotide (13 kDa) bound to M.SssI. As control, the unmodified complementary strand of ds-F duplex was radiolabeled (ds-F^#^ duplex) (Fig. 2B lane 10); and as expected, only the band with the lowest mobility was detected, corresponding to the enzyme covalently bound to the double-stranded DNA and suggesting that the enzyme binds to the modified strand. To confirm that the cross-link takes place on the F-modified strand, samples treated or not with proteinase K were loaded onto a denaturing sequencing polyacrylamide gel (Fig. S2). M.SssI was covalently bound only to the radiolabeled strand containing 5-F-CdR and after proteinase K treatment the degradation products of the protein attached to this strand appeared.

**Figure 3:**
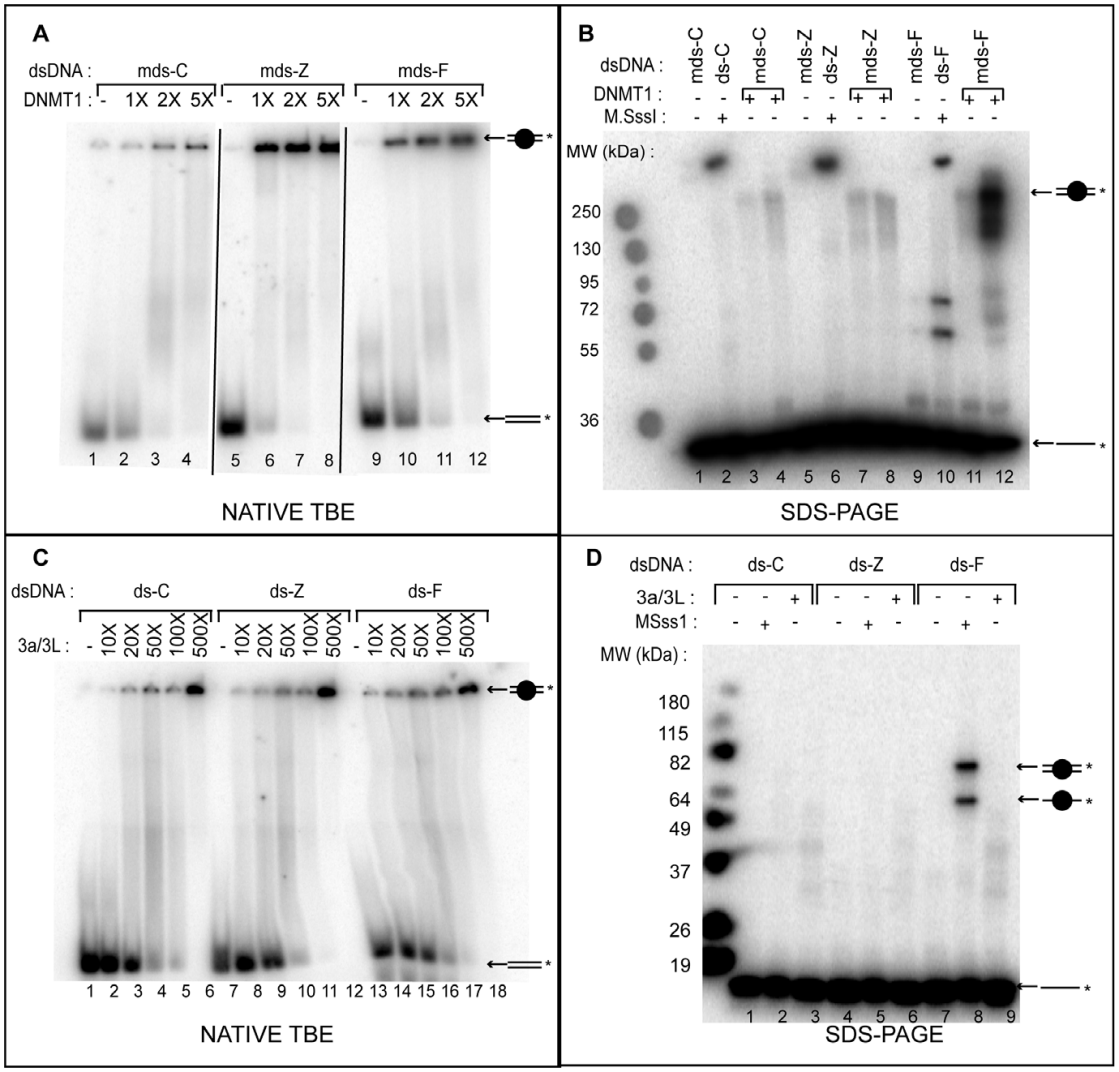
Native TBE and SDS-PAGE analysis of the binding and cross-linking between DNMT1 (A and B), DNMT3a/L (C and D) to DNA. M. S*ssI* was used as control. 5′ [γ^32^P]-labeled duplexes ds-C, ds-Z, ds-F (for M*Sss*.I and DNMT3a/3L) or mds-C, mds-Z, mds-F (for DNMT1) (0.05 pmol) were incubated with the specified methyltransferase. DNMT1 and DNMT3a/3L complex were incubated in buffer A supplemented by 300 µM of AdoMet; M.SssI was incubated in the buffer supplied by NEB supplemented with 300 µM of AdoMet and 100 µg/mL of bovine serum albumin (BSA). (**A**) DNMT1 was used in excess of once (1X), twice (2X) or 5 times (5X) compared to the duplexes. Incubations were carried out for 4 h at 37°C. Samples were loaded on a 4% native TBE gel. (**B**) mds-X were incubated with DNMT1 at 28 nM final concentration for 16 h at 16°C (lanes 3, 7 and 11) or 16 h at 30°C (lanes 4, 8 and 12). As control ds-C, ds-Z and ds-F (lanes 2, 6 and 10, respectively) were incubated qith 0.5 µL of M.SssI (ca. 0.1 mg/mL) 16 h at 16°C. Samples were loaded on a 10% SDS-PAGE gel. (**C**) DNMT3a/3L complex was used in excess of 10 (10X) to 500 (500X) compared to the duplexes. Incubations were carried out for 1 h at 37°C. Samples were loaded on a 4% native TBE gel. (**D**) ds-X were incubated with DNMT3a/3L complex at 0.75 µM (final concentration) or 0.5 µL of M.SssI (ca. 0.1 mg/mL). Incubations were carried out for 16 h at 30°C (for DNMT3a/3L) or 16°C (for M.SssI). The reaction mixture was loaded onto a 10% denaturing SDS-PAGE. Schematic representation of the labeled single-stranded DNA (––*) or ds ( = *) are indicated on the right of the gels and the circle represents the enzyme bound to labeled single-stranded or double-stranded DNA.

Next we analyze the nature of the binding between zebularine or 5-F-CdR and the mammalian DNMTs: human DNMT1 and the catalytic domain of murine DNMT3a complexed with the C-terminal domain of DNMT3L (3a/3L) (Fig. 3). Hemimethylated duplexes mds-C, mds-Z and mds-F are used for DNMT1 due to its higher affinity for hemimethylated DNA [48] (Fig. 3A and 3B). Hemimethylated duplexes are incubated with increasing concentrations of DNMT1 in the presence of 300 µM of AdoMet and loaded on a native TBE gel (Fig. 3A). The complexes are detected in the wells of the gel and, at lower concentrations of protein migrate as smears in the gel. Qualitatively DNMT1 binds to mds-Z duplex with higher affinity (about twice) than to mds-F and to mds-C. To determine if this binding is covalent, SDS-PAGE gels are realized in various conditions. No irreversible covalent complex is detected when DNMT1 is incubated 16 h at 16°C or 30°C, with mds-C (Fig. 3B, lanes 3, 4) and mds-Z (Fig. 3B, lanes 7, 8). The same results are obtained after 4 h at 37°C (data not shown). In contrast, a covalent complex is detected on mds-F with a mobility of approximately 266 kDa in accordance with the molecular weight of tagged DNMT1 (240 kDa) and the duplex (26 kDa) (Fig. 3B, lanes 11, 12). When the reaction is performed at 16°C we detect only a weak irreversible complex (Fig. 3B, lane 11).

Native and denaturing gels are also realized to analyze the interaction between the three duplexes (ds) and of the catalytic DNMT3a/3L (3a/3L) complex (Fig. 3C and 3D). In the native gel the incubation conditions (1 h at 37°C) are chosen to avoid total methylation of the ds-C duplexes. Complexes are detected in the wells with all three duplexes (Fig. 3C). DNMT3a/3L binds slightly better to the ds-Z duplex compared to the others. However, no covalent complexes are observed in denaturing conditions after 16 h at 30°C (Fig. 3D, lanes 3, 6, 9), or 2 h at 37°C, or 16 h at 16°C (data not shown).

### Methyl group transfer does not occur on zebularine

**Figure 4:**
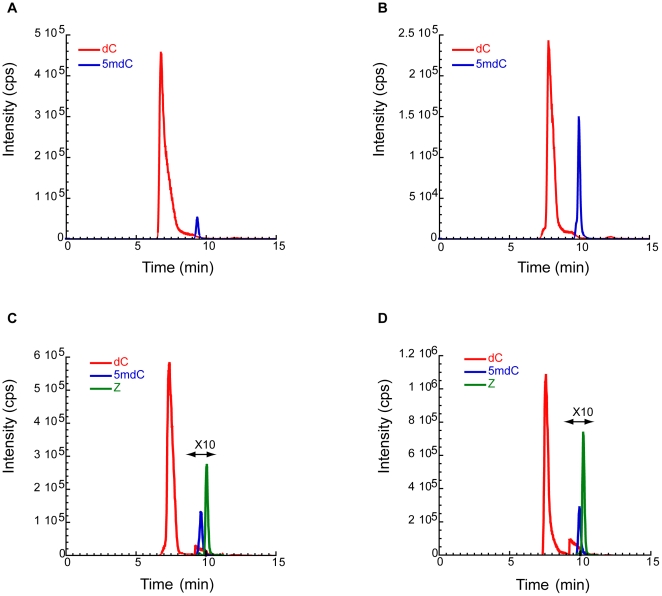
LC-ESI-MS/MS extracted ion chromatograms of 2′-deoxycytidine (dC, red line), 5-methyl-2′-deoxycytidine (5mdC, blue line) and zebularine (Z, green line). Chromatograms A and B show respectively nucleosides composition in ds-C before and after methylation by M.SssI. Chromatograms C and D show respectively nucleosides composition in ds-Z before and after methylation by M.SssI.

Another important issue is whether zebularine is methylated or not by the enzymes. To address this question, ds-Z or ds-C duplexes are incubated with or without M.SssI in the presence of 1 mM AdoMet 4 h at 37°C. Duplexes are then digested by nuclease P1, phosphodiesterase and alkaline phosphatase [49]. LC/MS-MS analysis in MRM mode is then used to detect the different nucleosides of the mixture (Fig. 4). We detected no C5-methylzebularine (Fig. 4D). Noteworthy, the technique is very sensitive (10 fmol). In addition, we observe that the enzyme does not methylate the other CpG sites present in the duplex when zebularine is present at one CpG. These results have been confirmed by a tritium-labeled AdoMet incorporation assay (data not shown).

Interestingly, AdoMet increases DNA enzyme binding and thus complex formation with ds-C, ds-Z and ds-F (Fig. S3). This suggests that the binding of the cofactor is important for complex formation, even if in the case of ds-Z no methylation occurs on the modified base.

### Increased stability of the ternary complex on DNA containing zebularine

**Figure 5:**
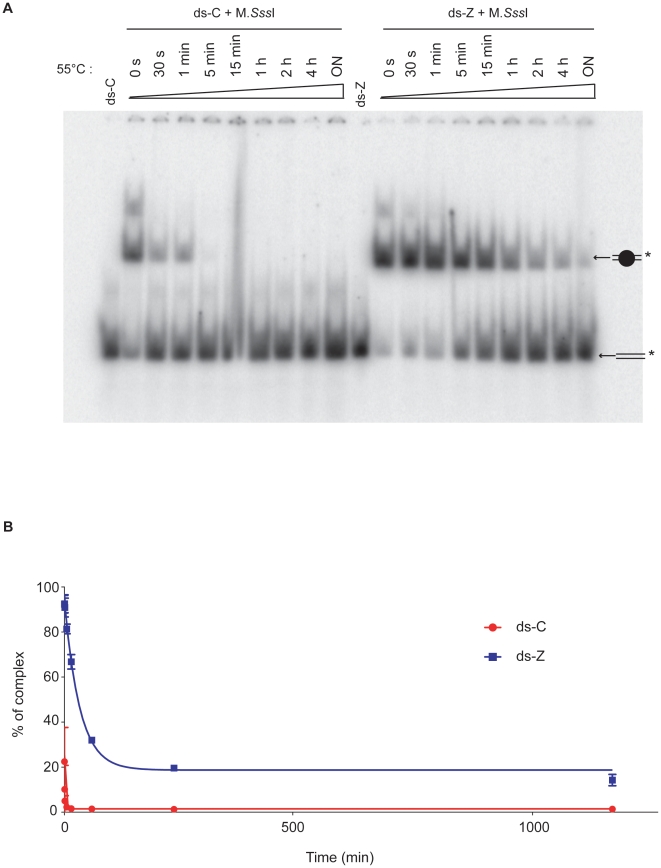
Dissociation analysis of the intermediate covalent complex. (A) ds-C or ds-Z were methylated by M.SssI during 16 h at 16°C before being incubated at 55°C during 0 sec, 30 sec, 1 min, 5 min, 15 min, 1 h, 2 h, 4 h and overnight. Lanes ds-C and ds-Z are controls and correspond to the duplexes loaded in the absence of the enzyme. The samples were run on a 10% non-denaturing gel. (B) Mean dissociation curves of 3 independent experiments for M.SssI/ds-C (red circles) and M.SssI/ds-Z (blue squares) complexes are reported with the standard error.

Next the dissociation of M.SssI complexed with ds-C, ds-Z and ds-F in the presence of AdoMet was studied by heat denaturing experiments. Such a method was chosen, because preliminary competition experiments with a 100-fold excess of unlabelled duplexes did not show any dissociation even after 4 hours (data not shown). In a first part of our study, we determined the temperature (among 20°C, 55°C, 75°C and 90°C) at which complex dissociation is observed without denaturing the duplexes. 55°C was chosen since dissociation is observed starting from 5 min of heating without dissociation of the duplex (Fig. 5A). Complexes are preformed by incubating duplexes with or without M.SssI in presence of 1 µM of AdoMet 16 h at 16°C. Samples are then subjected to heating at 55°C during various times from 30 s to overnight, before loading onto a native TBE gel (Fig. 5A). The ternary complex ds-Z/M.SssI/AdoMet is much more stable than the one formed with ds-C. The dissociation times (t_1/2_) are calculated from the mean dissociation curves of three independent experiments represented in Fig. 5B and we obtained 23±7 s for ds-C and 22±5 min for ds-Z, and higher than 2 h with a plateau at 30–40% of formed complex in the case of ds-F (Fig. S4).

## Discussion

Zebularine has been described as a potent inhibitor of DNA methyltransferases forming a permanent covalent complex with the enzymes [26], [27], [28]. Several studies have been carried out to clarify the mechanism of methylation inhibition by this nucleoside analog. It has been long debated why the covalent complex between DNA methyltransferases and zebularine containing DNA was permanent. Taylor *et al*. reported the formation of a covalent link between a short DNA duplex containing deoxyzebularine and M.MspI [27], showing however that these complexes are not as stable as those observed with a DNA containing 5-F-CdR. The authors extended their findings to M.HhaI [26] using the same technique of denaturing by SDS prior to loading onto a non-denaturing gel and observed that AdoMet and its analogs increase complex formation. Finally, in 2002, Zhou *et al*. described the interaction between the M.HhaI and a short duplex containing zebularine [28] by X-ray crystallography observing the formation of a covalent bond between the enzyme and zebularine. However, according to the authors, it remains unexplained why the covalent bond formed during the reaction with the enzyme and zebularine might be permanent, *i.e.* why the β-elimination to reverse adduct formation should not take place. A study using an electronic structure-based algorithm predicted the inhibition by noncytosine nucleotide targets [5]. Most recently, Darii *et al*. [46] showed that M.SssI formed SDS-resistant complex with DNA containing zebularine in presence of either AdoMet or AdoHcy by denaturing Laemmli polyacrylamide gel analysis. However samples were not heated before loading. Finally, van Bemmel *et al.* [44] analyzed the difference between 5-aza-CdR and zebularine. They report that oligonucleotides containing zebularine are competitive inhibitors of both prokaryotic (M.*Hha*I) and mammalian DNMT (DNMT1). They conclude that the covalent complex formed with the enzyme is irreversible with the oligonucleotide containing 5-azacytosine and reversible but stable for the one containing zebularine.

In this work, we studied the mechanisms of inhibition of zebularine compared to 5-F-CdR on prokaryotic M.SssI and mammalian DNMT 1 and DNMT3a/3L. We confirm that the ternary complex between DNA containing zebularine, AdoMet and the enzymes is reversible. In addition we clearly show that:

(i) the methyltransferases have a higher affinity for the zebularine-containing DNA than unmodified DNA or the 5-F-CdR-containing one. We measured the DNA binding of M.SssI for the three duplexes (Fig. 2A and S1) and we found that, although zebularine does not form a covalent complex with the DNMTs tested, it increases the binding affinity of the enzyme for the DNA by 2-folds.

Moreover in agreement with previous finding, AdoMet increases the affinity (Fig. S3B). Similarly, DNMT1 and DNMT3a/3L bind zebularine modified DNA with a higher affinity than unmodified and 5-F-CdR duplexes (Fig. 3A and 3C). However, in the case of hDNMT1 and murine catalytic DNMT3a/3L, the DNA/enzymes complexes are detected in the wells of the gel so it is difficult to do a quantitative analysis. We assume that this is because we use for our studies 40mer duplexes allowing the binding of several enzymes and thus the formation of high molecular weight complexes that do not enter into the gel. Yokochi *et al*. [50] have already performed gel shift analysis with hDNMT1 and mDNMT3a on 21 pb long duplexes, revealing multiple protein-DNA complexes, with 1, 2, 3 or 4 proteins bound to the duplex. The higher DNA binding of the methyltransferases for ds-Z can be explained by a reduction in the barrier to base flipping since the lack of the amino group on zebularine implies that the Z•G base pair is held by only 2 H bonds instead of 3 present in the unmodified C•G base pair. Moreover, the lack of 4-amino group causes a profound increase in the electrophilic character of the base compared with cytidine that facilitates the nucleophilic attack taking place at C6 and formation of the covalent intermediate [29], [32].

(ii) We have shown that both prokaryotic and mammalian DNMTs bind to the three duplexes by non denaturing gel analysis. However we demonstrate by denaturing gel analysis that the binding between zebularine containing DNA and the enzymes is reversible and heat labile. This finding implies that the covalent intermediate formed during the catalytic process between the catalytic cysteine and the C6 of the base is not permanent. Van Bemmel *et al.* showed that the complexes formed by DNMT1 and M.HhaI with DNA containing zebularine are stable in the presence of SDS at room temperature. However, theses complexes completely dissociated at 75°C for M.*Hha*I. For DNMT1 only a very small population of complex was still detectable at 95°C. The authors suggest that zebularine is trapped by a covalent bond formation with an unidentified electrophile in the catalytic pocket of the enzyme. In our experimental conditions, we did not observe such complexes. As control, we used 5-F-CdR-containing duplex that clearly forms an irreversible DNA-enzyme complex with M.SssI and DNMT1 detectable even after heating at 90°C (Fig. 2B and 3B). No such covalent complex is observed with the murine catalytic DNMT3a/3L complex, which can be explained by the difference in the activity between the enzymes. Indeed, M.SssI and DNMT1 are more active than DNMT3a/3L [47], [51]. Reither *et al*. have observed a covalent complex between DNA containing 5-fluorodeoxycytidine and the murine catalytic domain of DNMT3a [52]; however, the complex was very weak.

(iii) No methylation of zebularine is observed by LC-MS/MS analysis on digested ds-Z after incubation with M.SssI (Fig. 4). This result is in agreement with the molecular orbital calculations made by Zhou *et al.* [28], indicating that zebularine did not seem to be methylated. This explains also the observation of van Bemmel *et al.* [44] that the stability of M.*Hha I* complexed to zebularine-containing DNA was independent of the use of AdoMet or AdoHcy. In brief, our hypothesis is that the absence of 4-amino group in zebularine does not allow the activation of the cytosine C5 position after covalent intermediate formation and thus transfer of the methyl group.

(iv) The dissociation rate of ds-Z complexed to M.SssI is decreased of 57-folds compared to ds-C (Fig. 5B). It has been described that the DNA/DNMT complex dissociates rapidly in solution only after methylation of position 5 probably because of steric clash [45], [53]–[55]. Therefore the lack of methylation could explain the stabilization of zebularine-containing DNA binding complex as compared to the cytosine containing duplex ds-C. In addition, in our experimental conditions, when zebularine is present on the duplex, no methylation is observed even at the other CpG sites present on the duplex.

Thus, even if it is reversible, the lack of zebularine methylation seems to prevent the DNA/enzyme complex dissociation, and the zebularine-containing DNA/M.SssI/AdoMet ternary complex is much more stable than the one with unmodified DNA, indicating that the covalent intermediate is stabilized. In line with previous work [44], the fact that there is no permanent covalent bond with DNMTs may explain why higher concentrations of zebularine are needed to obtain similar levels of demethylation in cells in comparison with 5-aza-CdR [31] and why its cytotoxicity is lower.

## Materials and Methods

### General reagents

Oligodeoxynucleotides were purchased from Eurogentec and enzymes from New England Biolabs (NEB) or Roche Biochemicals, at the exception of DNMT1 (BPS Bioscience, CA) and catalytic murine DNMT3a and 3L, which were purified as described in [56]. [γ^32^P]ATP (3000 Ci/mmol) was purchased from Perkin Elmer and Micro Bio-Spin® 6 columns from Biorad.

### Nomenclature

ss stands for the single-stranded oligonucleotide containing either deoxycytidine (ss-C), zebularine (ss-Z) or 5-fluorodeoxycytidine (ss-F). When hybridized to the complementary strand, we obtain duplexes ds-C, ds-Z and ds-F. mds means that the duplex is hemimethylated, *i.e.* methylated on the complementary strand not containing the modification.

### Labeling and duplex formation

The indicated single-strand of the duplex was radiolabeled at its 5′ by T4 polynucleotide kinase and [γ^32^P]ATP according to the manufacture instructions. Then it was hybridized in 20 mM Tris pH 7.5, 10 mM LiCl with 1.4 excess of unlabeled complementary strand, heated at 90°C for 5 min and slowly cooled down to RT.

### Covalent complex formation

5′ [γ^32^P] labeled duplexes ds-X (for M*Sss*.I and DNMT3a/3L) or mds-X (for DNMT1) (0.05 pmol) were incubated with specified DNMTs at the concentration indicated in the figure legend: M.SssI in the buffer supplied by NEB; DNMT1 (0.05 mg/mL) and DNMT3a/3L complex in buffer A (20 mM HEPES pH 7.2, 1 mM EDTA, 50 mM KCl, 50 µg/mL BSA) supplemented with 300 µM of AdoMet. Incubations were carried out for 16 h at 16°C (for M.SssI) or as described in the figure legend. The reaction mixture was loaded onto a pre-run 4% non-denaturing polyacrylamide gel in TBE buffer or onto a 10% denaturing SDS-PAGE after boiling in Laemmli buffer. The gels were vacuum-dried and protein-DNA complexes were visualized on a Typhoon (GE Healthcare). All experiments were performed in replicate independently.

### Methylation status of zebularine

ds-Z (1 nmol) were incubated with M.SssI and then digested by nuclease P1 (2 h at 45°C in 0.01 M AcONH_2_ buffer), snake venom phosphodiesterase (2 h at 37°C in 0.1 M NH_4_HCO_3_ buffer) and antarctic alkaline phosphatase (1 h at 37°C) according to the procedure described by Song *et al.* [49]. Products were then analyzed by LC-MS/MS in MRM mode with the monitor of 4 transition pairs. dC, 5mdC and Z were detected respectively by monitoring *m/z* 228.2/112.2, *m/z* 242.2/126.2 and *m/z* 229.2/97.2. 5-methylzebularine was searched by monitoring *m/z* 243.2/111.2 but was not detected.

Alternatively, the methylation status was also tested in an *in vitro* radioactive methylation assay. DNA methylation activity of the complex is measured by the incorporation of tritiated methyl groups from labeled S-[methyl-^3^H] AdoMet (specific activity 370 GBq/mmol, Perkin Elmer) into the DNA substrates. The methylation reactions were carried out in the NEB methylation buffer, using 0.1 µM DNA, 0.5 µM of labeled AdoMet, 0.5 µM of non-labeled AdoMet and 0.5 µM of M.SssI. The methylation reactions were started by the addition of substrate DNA and allowed to proceed for 1 hour at 37°C. Reaction were stopped on a Micro Bio-Spin 30 column (BioRad) and measured in 4 mL of scintillate liquid.

All experiments were performed in duplicate or triplicate independently.

### Ternary complex dissociation studies

5′ [γ^32^P] labeled duplexes ds-X (for M*Sss*.I) (0.05 pmol) were incubated with or without M.SssI (1 µL of M.SssI (ca. 0.1 mg/mL)) in the buffer supplied by NEB supplemented with 1 µM of AdoMet and 100 µg/mL of bovine serum albumin (BSA). Incubations were carried out for 16 h at 16°C and samples were then heated at 55°C during various times from 30 s to overnight. The reaction mixture was loaded onto a pre-run 4% non-denaturing polyacrylamide gel in TBE buffer and protein-DNA complexes were visualized on a Typhoon (GE Healthcare). All experiments were performed in triplicate independently.

## Supporting Information

Figure S1 Binding analysis of the three duplexes with M.SssI on native TBE gels. 5′ [γ32P]-labeled duplexes on the stranded containing the modified base (ds-Z, ds-F and the control ds-C) (0.05 pmol) were incubated with or without various amount of M.SssI in the buffer supplied by NEB supplemented with 300 µM of AdoMet and 100 µg/mL of bovine serum albumin (BSA) for 16 h at 16°C. Concentrations of enzyme used were 23 nM, 69 nM, 92 nM, 115 nM, 161 nM and 230 nM for ds-C and ds-F and 11 nM, 23 nM, 34.5 nM, 46 nM, 57.5 nM, 69 nM, 92 nM and 115 nM for ds-Z. Samples were loaded on a 4% non-denaturing TBE gel. (A) Representative gel. Lanes ds-C, ds-Z and ds-F are controls and correspond to the duplexes loaded in the absence of the enzyme. Schematic representation of the labeled double-stranded DNA ( = *) is indicated on the right of the gel and the circle represents the enzyme bound to labeled double-stranded DNA. Bands of slower mobility correspond probably to multiple complexes. (B) Quantitative analysis of the above gel. Experiments have been replicated twice for ds-C and ds-F and four times for ds-Z to determine the mean of C50, concentration at which 50% of complex is formed, given in text.
(2.86 MB TIF)

Figure S2 Covalent complex formation with 5-fluorocytidine (5-F-CdR). (A) Denaturing gel analysis of the cross-linking between M.SssI and the duplex containing 5-F-CdR 5′ [γ32P]-labeled on the stranded containing the modified base (dsF) or on the on the complementary strand (ds-F#). ds-F or ds-F# are incubated with M.SssI at 16°C for 16 h and then treated with proteinase K (PK) 1 h at 37°C when specified on the top of the gel before loading onto a 8% polyacrylamide denaturing gel (7M urea and TBE 1X). Schematic representation of the labeled single-stranded DNA (––*) is indicated on the right of the gel and the circle represents the enzyme covalently bound to labeled single-stranded DNA. A band with retarded mobility appears only when the strand containing 5-F-CdR is labeled. Moreover when reaction mixtures are treated with proteinase K before loading, the retarded band disappears and products of enzyme degradation appear. These data show that M.SssI was covalently bound only on the modified strand. (B) Mechanism of covalent complex formation between 5-F-CdR and DNMT. Following covalent bond formation at position C6 and methyl transfer, the analogue remains bound to the active-site Cys, since β-elimination cannot be achieve.
(1.92 MB TIF)

Figure S3 Effect of AdoMet on the binding and cross-linking of M.SssI. 5′ [γ32P]-labeled ds-C (lane 1), ds-Z (lane 4) or ds-F (lane 7) were incubated with M.SssI, without (lanes 2, 5 and 8) or with AdoMet (lanes 3, 6 or 9) as mentioned on the top of the gel for 16 h at 16°C and loaded onto a 10% SDS-PAGE (A) or 4% non-denaturing TBE gel (B). The circle represents the enzyme bound to labeled double-stranded DNA. Schematic representation of the labeled single-stranded DNA (––*) or ds ( = *) are indicated on the right of the gel and the circle represents the enzyme covalently bound to labeled single-stranded or double-stranded DNA.
(1.13 MB TIF)

Figure S4 Dissociation analysis of the intermediate covalent complex between M.SssI and the 5-F-CdR-containing duplex (ds-F). (A) The duplex was incubated without M.SssI (lane ds-F) or with M.SssI during 16 h at 16°C before being incubated at 55°C during 0 sec, 15 min, 1 h, 4 h and overnight. The M.SssI/DNA complexes were analyzed on a non-denaturing gel. (B) Mean dissociation curves of 3 independent experiments for M.SssI/ds-F complexes is reported. Errors bars represent standard deviation. The graph is done with Prism software.
(2.08 MB TIF)
