# Supplementary figures and images for: Mechanistic Insights on the Inhibition of C5 DNA Methyltransferases by Zebularine
Source: PLoS One. 2010 Aug 24;5(8):e12388. doi: 10.1371/journal.pone.0012388 (PMC2927531; doi:10.1371/journal.pone.0012388)

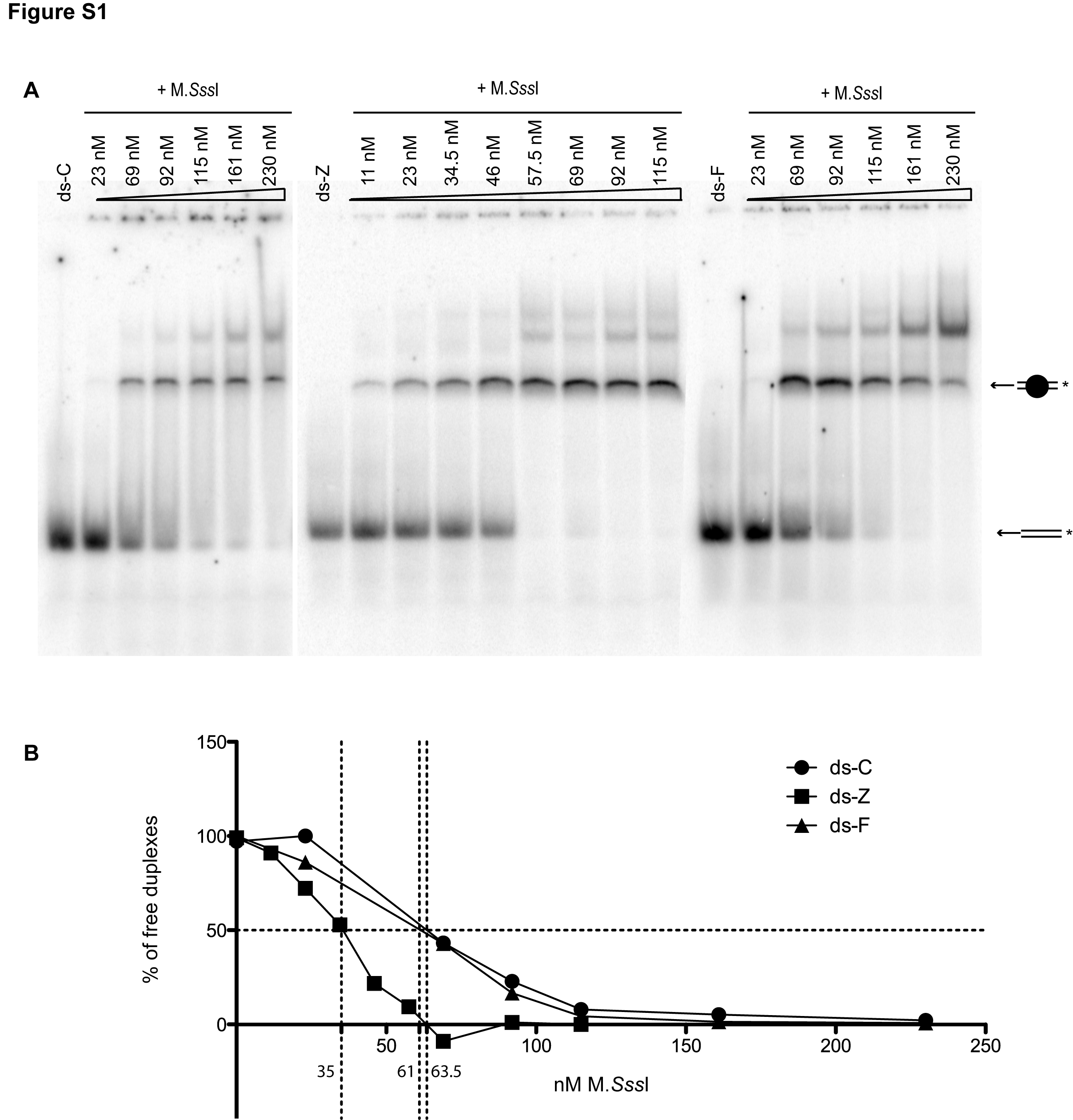

Supplement: Figure S1 — Binding analysis of the three duplexes with M.SssI on native TBE gels. 5′ [γ32P]-labeled duplexes on the stranded containing the modified base (ds-Z, ds-F and the control ds-C) (0.05 pmol) were incubated with or without various amount of M.SssI in the buffer supplied by NEB supplemented with 300 µM of AdoMet and 100 µg/mL of bovine serum albumin (BSA) for 16 h at 16°C. Concentrations of enzyme used were 23 nM, 69 nM, 92 nM, 115 nM, 161 nM and 230 nM for ds-C and ds-F and 11 nM, 23 nM, 34.5 nM, 46 nM, 57.5 nM, 69 nM, 92 nM and 115 nM for ds-Z. Samples were loaded on a 4% non-denaturing TBE gel. (A) Representative gel. Lanes ds-C, ds-Z and ds-F are controls and correspond to the duplexes loaded in the absence of the enzyme. Schematic representation of the labeled double-stranded DNA ( = *) is indicated on the right of the gel and the circle represents the enzyme bound to labeled double-stranded DNA. Bands of slower mobility correspond probably to multiple complexes. (B) Quantitative analysis of the above gel. Experiments have been replicated twice for ds-C and ds-F and four times for ds-Z to determine the mean of C50, concentration at which 50% of complex is formed, given in text. (2.86 MB TIF) [file pone.0012388.s001.tif]

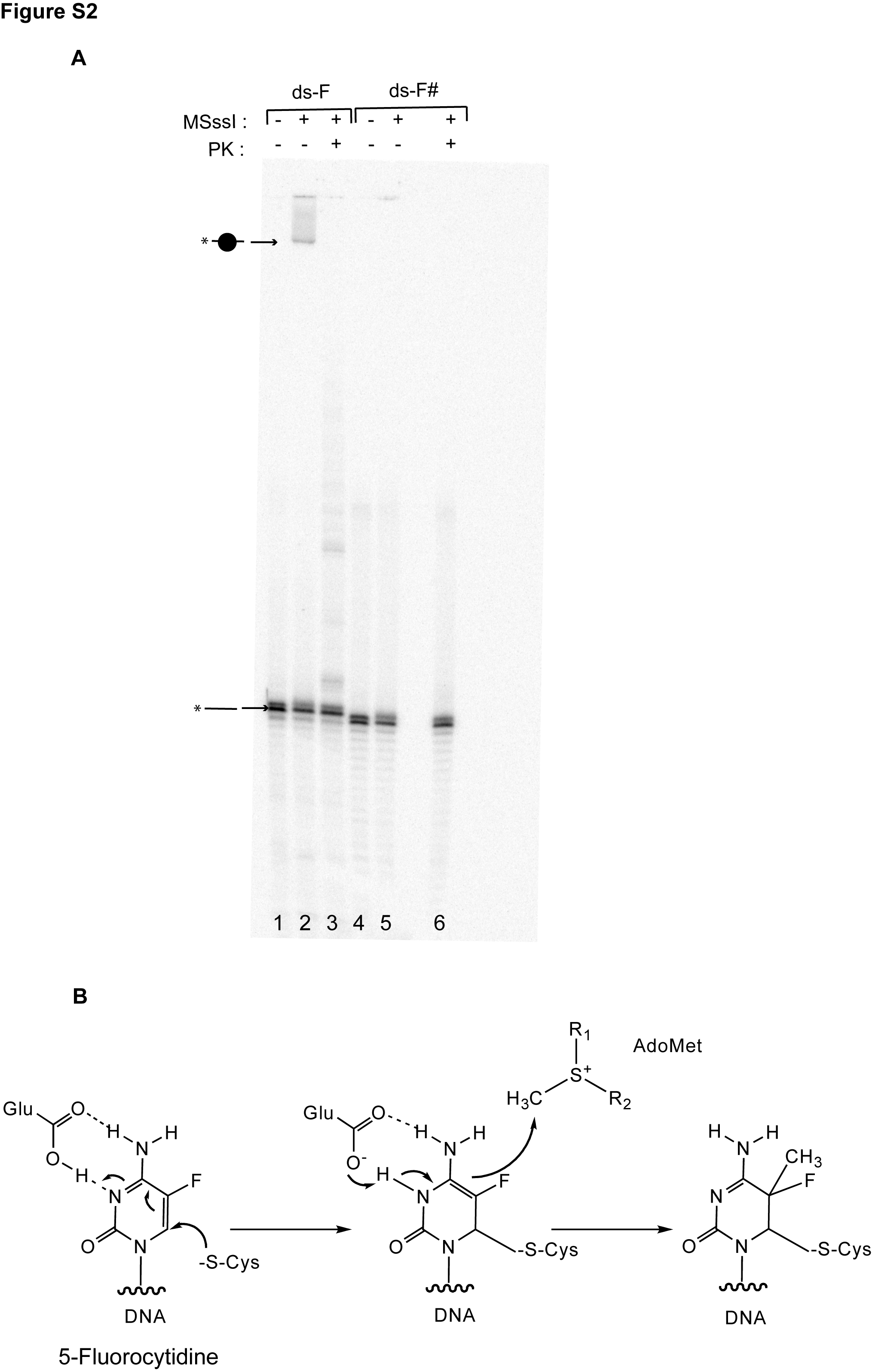

Supplement: Figure S2 — Covalent complex formation with 5-fluorocytidine (5-F-CdR). (A) Denaturing gel analysis of the cross-linking between M.SssI and the duplex containing 5-F-CdR 5′ [γ32P]-labeled on the stranded containing the modified base (dsF) or on the on the complementary strand (ds-F#). ds-F or ds-F# are incubated with M.SssI at 16°C for 16 h and then treated with proteinase K (PK) 1 h at 37°C when specified on the top of the gel before loading onto a 8% polyacrylamide denaturing gel (7M urea and TBE 1X). Schematic representation of the labeled single-stranded DNA (––*) is indicated on the right of the gel and the circle represents the enzyme covalently bound to labeled single-stranded DNA. A band with retarded mobility appears only when the strand containing 5-F-CdR is labeled. Moreover when reaction mixtures are treated with proteinase K before loading, the retarded band disappears and products of enzyme degradation appear. These data show that M.SssI was covalently bound only on the modified strand. (B) Mechanism of covalent complex formation between 5-F-CdR and DNMT. Following covalent bond formation at position C6 and methyl transfer, the analogue remains bound to the active-site Cys, since β-elimination cannot be achieve. (1.92 MB TIF) [file pone.0012388.s002.tif]

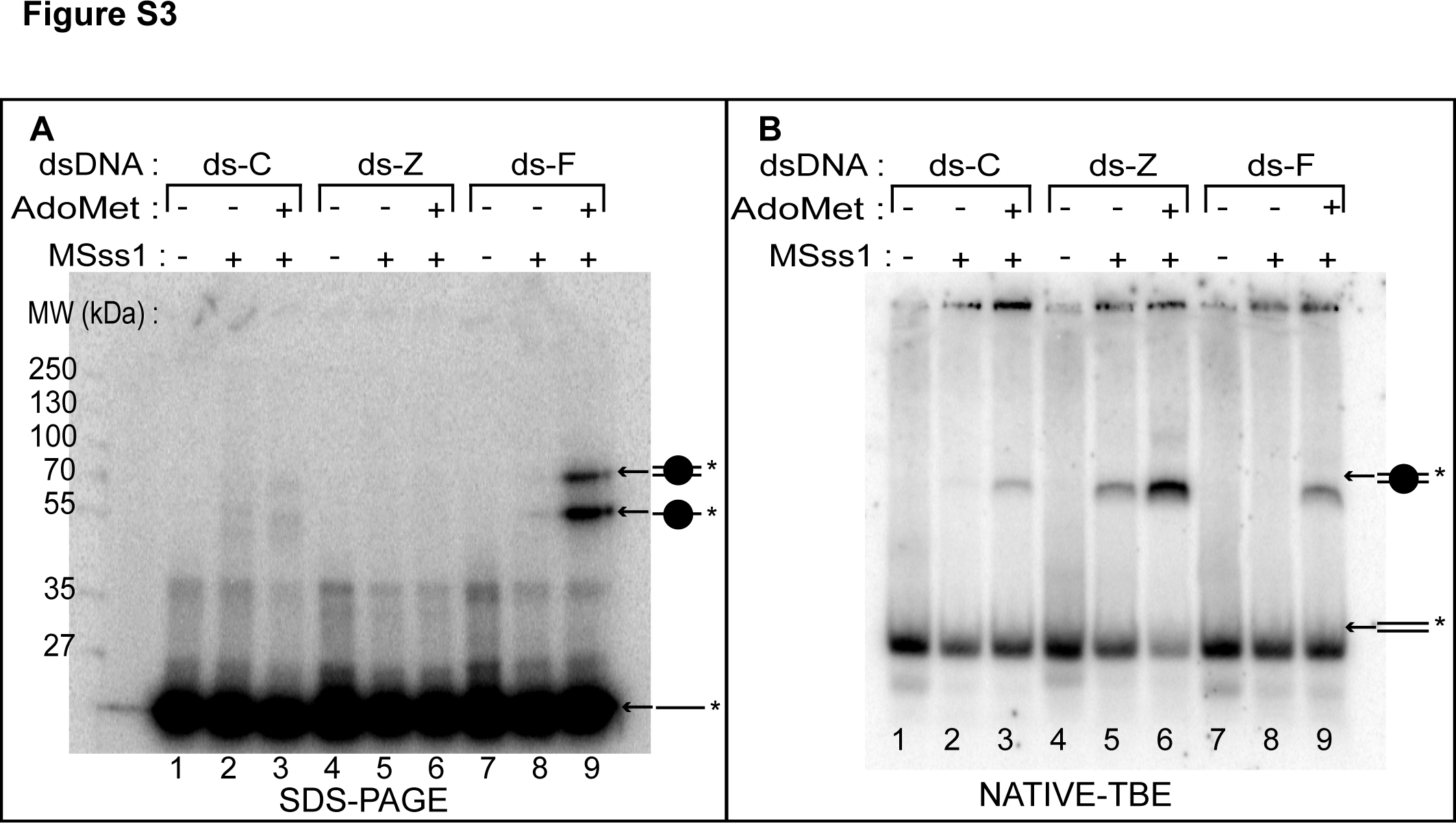

Supplement: Figure S3 — Effect of AdoMet on the binding and cross-linking of M.SssI. 5′ [γ32P]-labeled ds-C (lane 1), ds-Z (lane 4) or ds-F (lane 7) were incubated with M.SssI, without (lanes 2, 5 and 8) or with AdoMet (lanes 3, 6 or 9) as mentioned on the top of the gel for 16 h at 16°C and loaded onto a 10% SDS-PAGE (A) or 4% non-denaturing TBE gel (B). The circle represents the enzyme bound to labeled double-stranded DNA. Schematic representation of the labeled single-stranded DNA (––*) or ds ( = *) are indicated on the right of the gel and the circle represents the enzyme covalently bound to labeled single-stranded or double-stranded DNA. (1.13 MB TIF) [file pone.0012388.s003.tif]

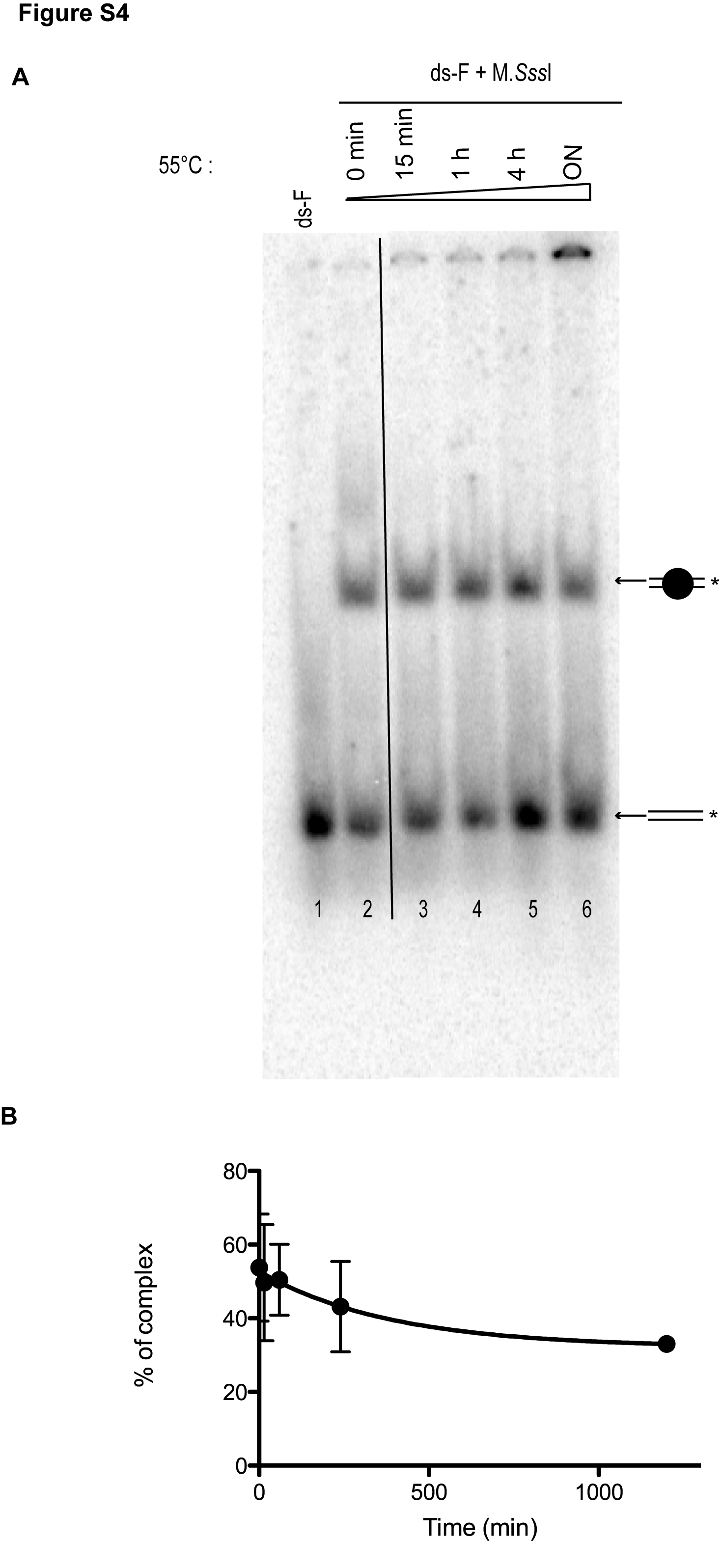

Supplement: Figure S4 — Dissociation analysis of the intermediate covalent complex between M.SssI and the 5-F-CdR-containing duplex (ds-F). (A) The duplex was incubated without M.SssI (lane ds-F) or with M.SssI during 16 h at 16°C before being incubated at 55°C during 0 sec, 15 min, 1 h, 4 h and overnight. The M.SssI/DNA complexes were analyzed on a non-denaturing gel. (B) Mean dissociation curves of 3 independent experiments for M.SssI/ds-F complexes is reported. Errors bars represent standard deviation. The graph is done with Prism software. (2.08 MB TIF) [file pone.0012388.s004.tif]
